# Digital Therapeutics for Improving Effectiveness of Pharmaceutical Drugs and Biological Products: Preclinical and Clinical Studies Supporting Development of Drug + Digital Combination Therapies for Chronic Diseases

**DOI:** 10.3390/jcm13020403

**Published:** 2024-01-11

**Authors:** Zack Biskupiak, Victor Vinh Ha, Aarushi Rohaj, Grzegorz Bulaj

**Affiliations:** 1 Department of Medicinal Chemistry, College of Pharmacy, University of Utah, Salt Lake City, UT 84112, USA; 2 The Spencer Fox Eccles School of Medicine, University of Utah, Salt Lake City, UT 84113, USA

**Keywords:** digital health, mHealth, smartphone app, self-management, self-efficacy, analgesic drugs, anti-cancer drugs, antidepressant drugs, antiseizure medication, anxiolytic drugs

## Abstract

Limitations of pharmaceutical drugs and biologics for chronic diseases (e.g., medication non-adherence, adverse effects, toxicity, or inadequate efficacy) can be mitigated by mobile medical apps, known as digital therapeutics (DTx). Authorization of adjunct DTx by the US Food and Drug Administration and draft guidelines on “prescription drug use-related software” illustrate opportunities to create drug + digital combination therapies, ultimately leading towards drug–device combination products (DTx has a status of medical devices). Digital interventions (mobile, web-based, virtual reality, and video game applications) demonstrate clinically meaningful benefits for people living with Alzheimer’s disease, dementia, rheumatoid arthritis, cancer, chronic pain, epilepsy, depression, and anxiety. In the respective animal disease models, preclinical studies on environmental enrichment and other non-pharmacological modalities (physical activity, social interactions, learning, and music) as surrogates for DTx “active ingredients” also show improved outcomes. In this narrative review, we discuss how drug + digital combination therapies can impact translational research, drug discovery and development, generic drug repurposing, and gene therapies. Market-driven incentives to create drug–device combination products are illustrated by Humira^®^ (adalimumab) facing a “patent-cliff” competition with cheaper and more effective biosimilars seamlessly integrated with DTx. In conclusion, pharma and biotech companies, patients, and healthcare professionals will benefit from accelerating integration of digital interventions with pharmacotherapies.

## 1. Introduction

A high prevalence of chronic diseases has challenged healthcare systems and public health [1]. The most effective way to reduce the impact of chronic medical conditions is to integrate disease management and prevention with pharmacological and digital health innovations [2]. Clinical benefits of pharmaceutical drugs and biologics are confronted by such limitations as drug-resistance, medication non-adherence, adverse effects, affordability, accessibility, and inadequate efficacy. Drug-related morbidities and mortality also contribute to increased healthcare spending [3]. Clinical benefits of digital health technologies are balanced by issues related to cybersecurity, privacy, engagement and attrition rates, the reimbursement process, evolving regulatory process, and rapid advances in technology that can outpace their implementation into health care. The pharmaceutical industry has embraced digital transformation, further accelerated by artificial intelligence (AI) [4,5].

DTx are mobile medical apps that have received the US Food and Drug Administration (FDA), or other regulatory agency, authorization for treating, or preventing, specific medical conditions through a “software as a medical device” (SaMD) regulatory pathway [5,6,7,8,9]. Since DTx are medical devices, their integration with drugs and biologics can follow the drug–device combination product guidelines. The FDA Office of Combination Products defines a combination product as “A product comprised of two or more regulated components, i.e., drug/device, biologic/device, drug/biologic, or drug/device/biologic, that are physically, chemically, or otherwise combined or mixed and produced as a single entity” [10]. To support development efforts towards marketing of drug + digital combination therapies, the FDA draft guideline, “Regulatory Considerations for Prescription Drug Use-Related Software”, describes regulatory solutions for integrating a mobile app with prescription drugs and biologics [11].

**Figure 1:**
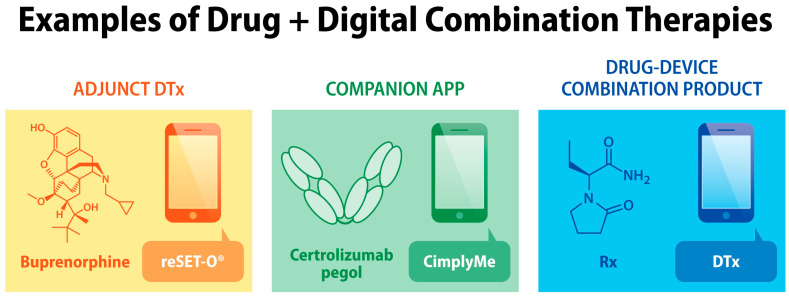
Examples of drug + digital combination therapies for the treatment of chronic diseases. The reSET-O mobile medical app for opioid use disorder (OUD) was authorized by the FDA in combination with buprenorphine. The CimplyMe companion app was designed for rheumatoid arthritis or Crohn’s disease patients who take an anti-TNFα biologic, certolizumab pegol. DTx delivering epilepsy self-management and music-based intervention was proposed as a drug–device combination product together with an antiseizure drug (levetiracetam is shown as an example) [13].

Combining clinical benefits of drug- and digital-based therapies can outweigh their limitations, while simultaneously offering personalized therapies for people living with chronic diseases [12,13,14,15]. For example, the FDA authorization of reSET-O, an adjunct DTx in combination with buprenorphine for opioid use disorder, illustrates one strategy to create drug + digital combination therapies [16,17]. The use of reSET-O in combination with buprenorphine significantly increased opioid abstinence and treatment retention [18]. Digital interventions improve opioid-based analgesia [19] and medication adherence [20,21]. Digital platforms delivering disease self-management and remote patient monitoring (e.g., Huma^®^, BlueStar^®^, Propeller^®^, HelloBetter^®^) offer means to improve pharmacotherapy outcomes via drug + digital combination therapies. Diverse combinations of digital health technologies and pharmacological treatments are illustrated in Figure 1.

Creating drug + digital combination products was proposed to improve control of seizures in people with refractory epilepsy, and to increase the value proposition of branded and generic drugs by expanding their intellectual property protection [13]. The design of a DTx prototype for epilepsy to be combined with an antiseizure medication (ASM) as a drug–device combination product illustrated a means to decrease dosing of a pharmaceutical drug without compromising clinical efficacy [22]. Sverdlov and colleagues discussed how drug + digital combination therapies can increase clinical efficacy of pharmacotherapies [12]. Development of drug + digital combination therapies can be accomplished through a two-step process: (1) development of DTx using the “software as a medical device” regulatory pathways (e.g., 510k clearance, de novo, premarket authorization (PMA)), and (2) development of DTx-Rx combination product, whereas DTx is a medical device.

For preclinical studies, our group described an approach to evaluate DTx “active ingredients” (audiogenic stimulation, cognitive stimulation, physical activities) in combination with pharmaceutical drugs [23], and proposed a preclinical strategy to evaluate drug + digital combination therapies in animal models of human diseases, using environmental enrichment (EE) as a surrogate for digital interventions (Table 3 in [23]). While research studies show clinical and cost-effectiveness benefits of digital interventions for diverse chronic conditions, to the best of our knowledge, there are no published studies on integrating pharmacological and digital interventions via drug + digital combination products [14,19,24,25].

In this narrative review, we summarize preclinical studies on EE and DTx “active ingredients”, as well as clinical studies on digital interventions across selected examples of chronic diseases, namely Alzheimer’s disease, dementia, rheumatoid arthritis, cancer, chronic pain, depression and anxiety, and epilepsy. A rationale for randomly choosing these diverse neurological, neurodegenerative, mental, inflammatory, and autoimune conditions was to review evidence supporting development of drug + digital combination therapies as a universal strategy for treating chronic disorders. A keyword-based search in PubMed, Google Scholar, and Embase databases was performed by three authors to identify systematic reviews, meta-analyses, randomized controlled trials, and preclinical studies on DTx-compatible interventions and EE. Each section of this review is organized by a specific chronic disease and provides examples of clinical effects of digital interventions, followed by preclinical evidence of EE and individual non-pharmacological modalities as a surrogate for DTx “active ingredients”. We also discuss the impact of drug + digital combination therapies on the innovation of generic drugs and biosilimars, drug repurposing, and gene therapies.

The main objective of this review is to encourage translational research on drug–device combination products. Herein, we summarize preclinical and clinical studies that bridge pharmacological and digital interventions. The focus on preclinical studies relevant to testing DTx “active ingredients” highlights novel approaches to improve drug-discovery outcomes when evaluating investigational new drug (IND) candidates. The focus on reviewing clinical studies of digital health technologies and DTx-compatible non-pharmacological interventions for chronic diseases highlights new opportunities for pharma/biotech companies and patients to increase clinically meaningful benefits via drug + digital combination therapies.

## 2. Alzheimer’s Disease and Dementia

Alzheimer’s disease (AD) is a progressive neurodegenerative disease that can lead to dementia. As AD and dementia progress, patients and caregivers are burdened with an increased demand for managing and providing care [26]. The slow progress towards effective pharmacological treatments for AD was recently disrupted by the FDA approvals of monoclonal antibodies (mAbs) such as aducanumab and lecanemab intended to reduce amyloid-β in the brain [27,28,29]. Another biologic, donanemab, is also expected to receive regulatory approval [30,31,32,33]. These new biologics add to a repertoire of pharmacological agents for AD and dementia, such as cholinesterase inhibitors (donepezil, rivastigmine, and galantamine) and NMDA antagonists (memantine). More drug candidates against AD are currently undergoing clinical trials [34].

From wearables that monitor physical and mental health to video games that improve cognitive functions, digital health technologies can improve therapy outcomes for AD and dementia patients [9,35,36,37,38,39]. One example of a mobile app for dementia patients is iWander which delivers audible prompts and improves patient–caregiver communications [40]. Another example is Backup Memory, a mobile app developed by Samsung for AD patients where patients go through daily reminders of past events to help slow down the progression of their disease. The WhatMatters app provides personalized support for dementia patients through caregivers [41]. Recommended mobile apps for people with AD and dementia were reviewed elsewhere [42,43,44].

**Figure 2:**
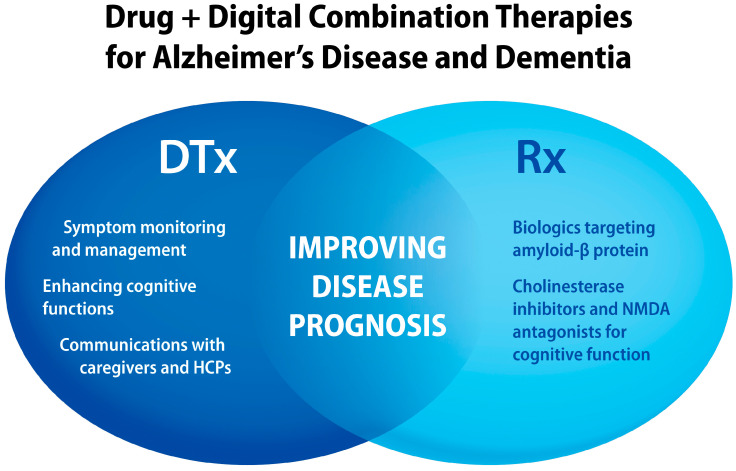
Examples of clinical and patient care benefits delivered by digital health technologies that can be combined with specific pharmacological agents targeting cognitive functions in AD and dementia patients. HCPs, healthcare professionals; NMDA N-methyl D-aspartate.

Research studies on digital interventions such as virtual reality (VR) and mobile apps for people with AD and dementia support patient care benefits [45,46,47,48,49]. Systematic review and meta-analysis (SR/MA) studies suggest that VR interventions can improve cognitive functions and ability to perform daily activities in AD patients with mild cognitive impairment (MCI) [45,49]. In addition to therapeutic effects [50], digital interventions can be useful for diagnosis, monitoring AD prognosis [47], improving communications [46], and preventing loneliness and social isolation [51]. Promises and challenges of digital health technologies for older people have been recently reviewed [38]. Since non-pharmacologic interventions, such as physical exercises and music, offer clinical benefits for AD and dementia patients [52,53,54,55,56,57], a combination of these modalities with pharmacotherapies can further improve patient care. Benefits of integrating digital and pharmacological interventions are summarized in Figure 2.

**Figure 3:**
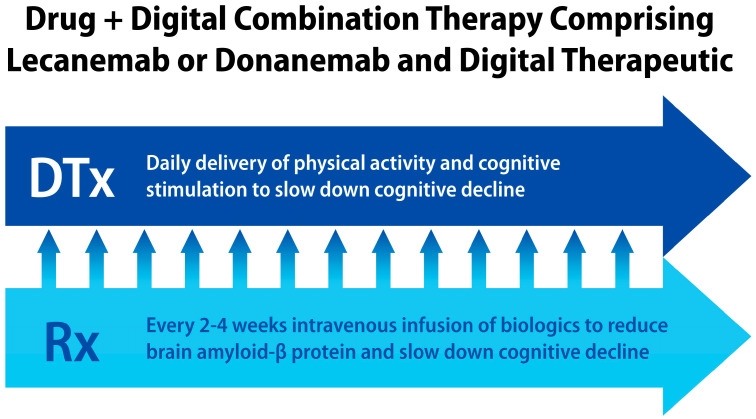
Drug + digital combination therapies for AD and dementia can include daily digital interventions between intravenous infusions of amyloid-β targeting monoclonal antibodies. Currently, the drug-alone therapies using lecanemab and donanemab require IV infusions every 2–4 weeks, thus missing daily opportunities of receiving clinically beneficial and personalized digital interventions to further improve therapy outcomes.

As illustrated in Figure 3, integration of digital and pharmacological therapies for AD patients can address some disadvantages of mAbs targeting amyloid-β protein, such as requiring intravenous administration (IV) every 2–4 weeks, limited efficacy, and the development of amyloid-related imaging abnormalities (ARIA) [29]. In between IV infusions, AD patients taking these biologics would benefit from digital interventions delivering non-pharmacological treatments, similar to those AD patients who received NMDA antagonists, acetylcholine esterase inhibitors, and internet-delivered multimodal treatments [58]. Daily digital interventions could include daily physical exercises, listening to music, and cognitive stimulation activities.

Animal studies in AD and dementia models showed that EE and physical activity improved spatial and working memories, and reduced levels of amyloid-β and tau proteins [59,60]. EE also improved cognitive functions in vascular dementia rats [61,62], and had positive effects on the cognitive reserve [63]. Zhang and colleagues [64] discovered that voluntary physical exercise ameliorated cognitive impairment in transgenic male APP/PS1 and wild-type mice. The running group had significantly shorter escape latency, better discrimination in the new object recognition test, and lower amyloid plaque deposition than sedentary AD mice. This finding is in accord with another study showing that both physical activity and cognitive stimulation can restore spatial memory, recognition, and motor deficits in the Tg4-42 AD mouse model [60]. Alzheimer’s rats that had both EE and donepezil showed significant improvement in performance on the Morris Water Maze tests compared to having either EE or donepezil alone, or neither [65].

**Figure 4:**
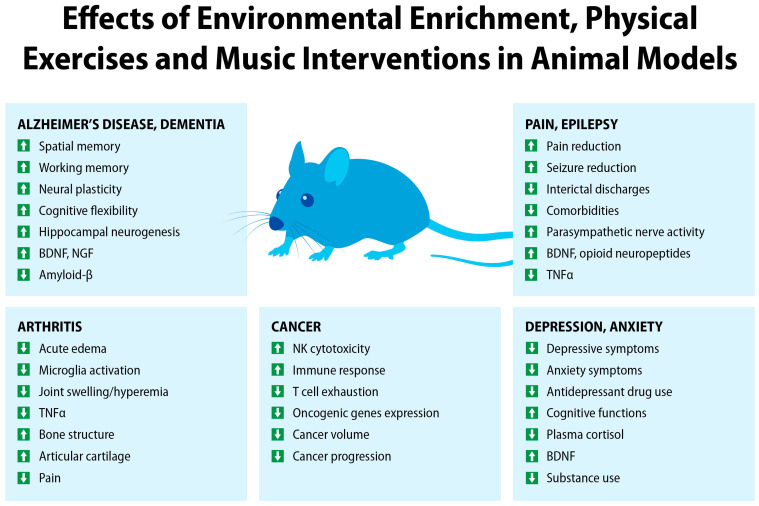
An overview of physiological effects of environmental enrichment and non-pharmacological interventions in animal models of human diseases. Upward arrows indicate an increase and an improvement. Downward arrows indicate a decrease.

Preclinical studies of the FDA-approved drugs for AD such as acetylcholinesterase inhibitors and NMDA antagonists can reduce cognitive decline and levels of amyloid-β protein in AD animal models [66]. Based on the effects of EE, testing drug-like compounds in the context of physical exercise, cognitive stimulation, music, and social interactions can further improve therapy outcomes, as compared to testing compounds under “standard” conditions. For example, EE and physical exercise increase neural plasticity, spatial and working memory [59], improve cognitive flexibility [67], increase hippocampal neurogenesis and expression of brain-derived neurotropic factor (BDNF) and nerve-growth factor (NGF) [68], and even reverse cognitive decline [63]. As illustrated in Figure 4, such EE-based preclinical studies may accelerate discovery and development of drug + digital combination therapies comprising non-pharmacological interventions with drugs targeting neurodegenerative pathways.

## 3. Rheumatoid Arthritis

Rheumatoid arthritis (RA) is a chronic disease that causes painful joint swelling and inflammation. Current pharmacological drugs used for arthritis include nonsteroidal anti-inflammatory drugs (NSAIDs), corticosteroids, and disease modifying anti-rheumatic drugs (DMARDs) [69]. Two main objectives of the therapy are pain relief and slowing the progression of joint damage [70]. NSAIDS are important for arthritis pain management [71], while DMARDs are immunomodulators (e.g., methotrexate and biologics such as certolizumab and adalimumab). Biological DMARDs can differ in their effectiveness [72,73], and can also increase the risk of serious infections [74].

Arthritis self-management programs improve therapy outcomes [75], and can be transformed into digital interventions [76,77,78,79,80,81]. Mobile apps for people living with RA (e.g., CareHand, LiveWith, The RAISE, RA Healthline, and ArthritisPower) differ in their content and quality [82,83,84,85]. These apps aim to improve patient outcomes with a variety of methods, such as tracking disease progression, providing patient education, encouraging healthy habits like physical exercise, and promoting social interactions and better nutrition. Users of the application LiveWith had higher scores on the patient self-efficacy of managing symptoms (P-SEMS) scale [86]. Patients with higher P-SEMS scores also tended to have lower levels of pain and increased levels of patient activation. Rodriguez and colleagues conducted a trial with the CareHand app, which included personalized exercise regimens, social functions, and patient education [80]. In their study, 53% of patients were also receiving concurrent drug treatments. Their findings showed that the group using the app + drug combination fared significantly better in their recovery compared to the group provided only with an exercise program. Despite promising clinical studies, real-world acceptance and adoption of digital interventions for RA is challenging [87,88].

**Figure 5:**
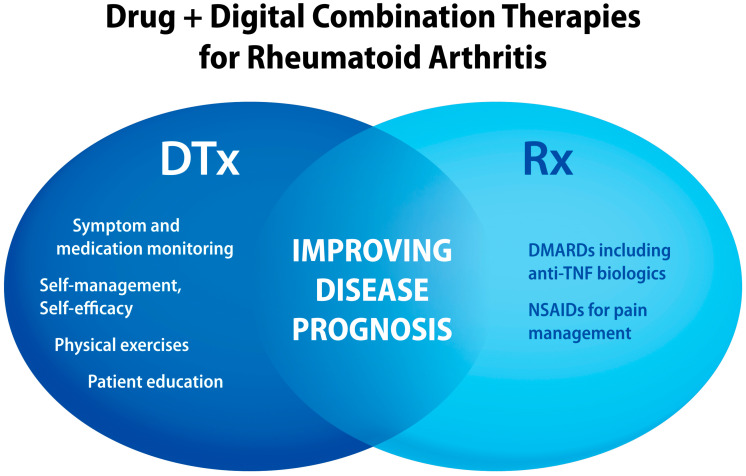
Examples of “active ingredients” delivered by digital health technologies that can be combined with DMARDs and analgesics to improve RA therapy outcomes. DMARDs, disease-modifying antirheumatic drugs; TNF, tumor necrosis factor.

Given that non-pharmacological interventions like physical exercise, quality sleep, optimized nutrition, social interactions, and patient education can improve patient outcomes, the combination of these methods with NSAIDs or DMARDs could further compound patient benefits [89,90]. Non-pharmacological management of pain, fatigue, inflammation, disability, and mental comorbidities is recommended for difficult-to-treat RA patients [89,91]. As an example of clinical benefits of combining pharmaceutical drugs with non-pharmacological interventions, a recent SR/MA suggested that exercise therapy was a better treatment option than NSAIDs and opioid analgesics for knee osteoarthritis pain [92]. As illustrated in Figure 5, integrating digital interventions with pharmacotherapies offers personalized therapies that aim to improve disease prognosis, as compared to “drug-alone” treatments.

Improving drug efficacy in animal models of arthritis can be achieved by testing compounds in the presence of EE (Figure 4). A number of preclinical studies in animal model of arthritis evaluated the effects of physical exercise [93,94,95,96]. Arthritic mice treated with exercise showed slower disease progression, thicker knee cartilage, and lower TNF-α levels compared to a control group [94]. The benefits of physical exercise on joint pathophysiology were reviewed by Derue and Ribero-da-Silva [93]. Running wheels, treadmills, or other exercise-based interventions in animal models improved preserved bone structure, downregulation of inflammatory signaling, improvement in weight asymmetry, and reduced pain compared to sedentary mice [93]. EE in the form of larger cages, running wheels, toys, and other enrichments ameliorated inflammatory changes, reduced acute edema, and increased expression of BDNF in the hippocampus among arthritic mice [97]. Preclinical studies showing positive effects of EE in animal arthritis models support EE-enhanced testing of new pharmacological compounds to improve the therapeutic window of potential IND candidates.

## 4. Cancer

Cancer is a chronic disease characterized by abnormal cells dividing uncontrollably and impacting healthy parts of the body. In addition to killing cancer cells, oncology patients often need to navigate pain, changes in their daily habits, mental and physical fatigue, and other symptoms related to both cancer and anti-cancer therapies. Current treatments for cancer include chemotherapy, immunotherapy, surgery, radiation therapy, hormone therapy, and cryoablation. For patients and healthcare professionals, the main clinical challenges are treatment adherence, symptom monitoring, symptom management, social support, and self-efficacy. Empowering oncology patients using digital technologies has been recognized as a promising strategy to improve therapy outcomes [98,99,100,101,102,103,104].

An early example of digital interventions for oncology patients is Re-Mission, a video game developed by Hope Labs that was shown to increase treatment adherence, cancer self-efficacy, and knowledge of cancer among younger patients [105,106]. Another example is an exercise-empowerment video game “Empower Stars”, which aimed to support children with cancer undergoing chemotherapy [107]. For adult oncology patients, a “LivingWith^®^” app delivers self-management interventions that reduced medical office visits [101]. Kaiku Health is a digital patient monitoring platform that supports cancer care, where patients can report symptoms, connect with their healthcare team, and receive self-care instructions to help detect cancer signs, symptoms, and relapses. This technology was also used to collect patient-reported outcomes during chemotherapy treatments [108,109,110]. It is noteworthy that the use of a web-based app to monitor symptoms and initiate palliative care significantly increased survival of lung cancer patients [111].

In a narrative review, Gussoni and colleagues summarized commercially available DTx for oncology indications [112]. The majority of these mobile apps are focused on symptom monitoring and management, and improving quality of life (QoL). Digital interventions can improve psychological outcomes [113], adherence to chemotherapy [106,114], and cancer pain management [115,116]. For example, Pain Guard app offers medication reminders, patient education, and treatment with the use of soothing music [117]. Using this app was associated with increased instances of pain remission, improved medication adherence, and reduced breakthrough pain [117]. Similarly, VR-based applications can reduce pain, fatigue, depression, and anxiety among cancer patients [118,119,120].

Mobile apps promoting physical activity can improve cancer-related fatigue, sedentary lifestyle, and psychosocial outcomes [121], also through personalized home exercise programs [122,123]. As cancer mortality declines, digital interventions delivering physical exercise interventions for cancer survivors are of equal importance [124]. A mobile app iCanFit was designed for cancer survivors to facilitate physical activity by tracking goals, finding resources, and providing peer-support and health education [125]. After 2–3 months of using iCanFit, the treatment group showed a significant increase in QoL and engagement in physical exercise [126]. Digitally delivered, personalized exercise programs, additionally supported by online health education, improved physical health among cancer survivors [127].

**Figure 6:**
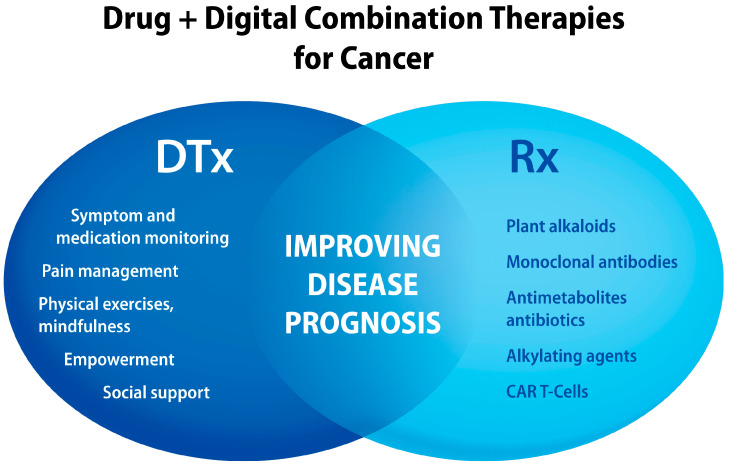
Examples of “active ingredients” delivered by digital health technologies that can be combined with antineoplastic drugs to improve cancer treatment outcomes. CAR T-cells, chimeric antigen receptor.

As illustrated in Figure 6, combining digital interventions with chemotherapy and immunotherapy agents is a rational strategy to improve cancer prognosis. For example, integrating cancer-specific DTx with pembrolizumab (Keytruda^®^) or paclitaxel as a drug–device combination product can offer more personalized treatments that maintain anti-cancer effectiveness, reduce drug side effects and cancer pain, improve psychosocial outcomes and health-related QoL, and support overall cancer care including communications with HCPs.

Animal models of cancer provide an opportunity to accelerate preclinical development of drug + digital combination therapies by testing anti-cancer drug candidates in the presence of DTx “active ingredients”. Studies show that EE in the form of physical exercise, social interactions, and cognitive and sensory stimulation can enhance anti-tumor immunity, increase lifespan, reduce tumor volume and cancer progression, and decrease cancer pain and chemotherapy-related toxicity [128,129,130,131,132]. The effects of physical exercise on cancer growth and treatment efficacy are generally positive [133,134]. Physical exercise was shown to enhance anti-PD-1 immunotherapies [135,136] and the efficacy of checkpoint inhibitors [137], and reduce doxorubicin-mediated cardiotoxicity in mice [138]. Stretching exercises for 10 min every day for 4 weeks in breast cancer mice models significantly reduced tumor volume and growth, as compared to the control group [139]. Kutz and colleagues discussed an exercise-oncology strategy to improve cancer treatments [136].

The promise of EE to improve cancer therapies is illustrated by an increased lifespan in colon cancer mouse model [129]. EE in the form of cages with running wheels, toys, and social interactions slowed tumor size and growth in pancreatic cancer mice [132]. Even simpler EE conditions such as providing an ‘igloo’ in the mice’s cage increased the NK cytotoxicity against Yac-1 lymphoma cells and decrease the number of tumors [131]. EE intervention in lung cancer mice reduced metastasis while increasing the number of lung-infiltrating NK cells and T and B lymphocytes [140]. EE can also include sensory stimulation, e.g., exposure of rats with bone cancer to music for two weeks showed lower tumor volumes and pain scores [128]. Music was also shown to mitigate a stress-induced increase in metastatic nodules in lungs of rats injected with carcinosarcoma cells [141]. These preclinical studies suggest that testing novel anti-cancer compounds in the presence of EE can increase their efficacy and decrease toxicity, thus widening their therapeutic window.

## 5. Chronic Pain

Chronic pain is defined as “pain that persists or recurs for more than 3 months” [142]. It is estimated that 25–30% of the human population is affected by pain [143,144], while inadequate pain treatment can lead to disability, mental health comorbidities, substance use disorder, and public health crisis [145,146,147]. NSAIDs, opioids, muscle relaxants, and other analgesic drugs are common pharmacological treatments for chronic pain. However, these medications provide short term pain relief, while causing adverse effects, gastrointestinal and cardiovascular toxicities, tolerance, and addiction. Non-pharmacological treatments for pain include physical exercise, psychological therapies, mindfulness and meditation, music, education, self-management, digital interventions, and other multimodal treatments [148,149,150,151,152,153,154]. A multimodal approach that integrates pharmacotherapy and non-pharmacological interventions enables more efficient and personalized pain management [14,24,155].

Since pioneering efforts to develop a VR-based technology for burn pain [156,157,158], DTx, such as RelieVRx, Kaia Health, and Hello Better Chronic Pain have expanded pain indications to other chronic conditions [24,159,160,161]. Digital therapeutic programs such as RelieVRx and Hello Better Chronic Pain are multi-week digital interventions that deliver patient education, mindfulness- and distraction-based practices, immersive environments, relaxation, breathing and physical exercises. RelieVRx received FDA authorization to market this prescription adjunt DTx treatment to adults with moderate to severe chronic low back pain, while Hello Better Chronic Pain is CE-certified as a medical device and DiGA-approved prescription app available in Germany. Kaia Health mobile technology can analyze body movements and recommend personalized physical therapy, as well as offering patient education, relaxation techniques, and consultations with coaches and medical providers. The Kaia Health app was shown to reduce non-specific lower back pain [159,162] and improve sleep in back pain patients [163]. This DTx is indicated for musculoskeletal pain, and is available in the US and Europe.

Clinical studies confirm the effectiveness of VR and mobile apps for acute, perioperative, and chronic pain [9,154,164,165,166,167,168,169,170]. These technologies deliver such “active ingredients” as physical exercises, psychotherapies, education, relaxation, self-management, and empowerment, while offering the convenience of at-home use [160]. Early post-marketing studies suggest an overall safety profile with a very low rate of adverse effects [171]. Challenges in developing DTx for chronic pain include meeting such primary care needs as patient–provider communications and counseling [172].

**Figure 7:**
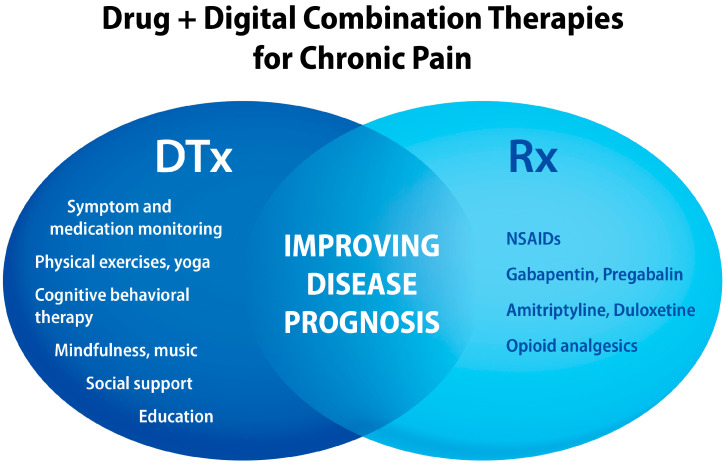
Examples of “active ingredients” delivered by digital health technologies that can be combined with analgesics to improve pain relief and chronic pain management. NSAIDs, nonsteroidal anti-inflammatory drugs.

The benefits of integrating digital interventions with analgesic drugs are illustrated in Figure 7. Of particular importance for patients taking opioids are DTx that can lead to drug tapering [173,174,175]. Since patient education is gaining recognition as an “active ingredient” for pain relief and management, digital technologies are being explored to scale up such interventions [160,176,177]. Given the analgesic properties of music [149,150,178,179,180,181,182], this non-pharmacological modality is underutilized as an adjunct digital intervention [183,184]. The compatibility of DTx with other pharmacological and non-pharmacological treatments as drug + digital combination therapies for chronic pain was highlighted in Figure 6 and Figure 7 in the perspective article [24].

For preclinical studies on drug + digital combination therapies for pain, our group proposed the use of EE as a surrogate for testing DTx “active ingredients” in combination with analgesic drugs [23]. In the carrageenan model of inflammatory pain in mice, the sensory stimulation (3-week exposure to music) significantly enhanced ibuprofen-based analgesia [23]. In the music-treated mice, cannabidiol and galanin-based NAX-5055 significantly reduced paw edema, suggesting positive interactions between the stimuli and drug treatments [23]. Music-induced analgesic effects were reported in a rat model of bone cancer pain [128], while other studies with mice produced mixed results [185,186,187]. Analgesic activities of physical exercises in rodents were reviewed elsewhere [188]. Another non-pharmacological modality tested in animal pain models is the exposure to specific light [189,190,191]. A light-emitting diode (LED) producing green light elicited antinociceptive effects in both neuropathic pain and postsurgical pain models in rats [189,191]. The light-induced analgesia was mediated by a release of endogenous opioid neuropeptides and reduced neuroinflammation [191,192]. The authors emphasized translational aspects of their findings to improve pain relief and reduce opioid use [191].

The effects of EE in animal pain models are well documented [193,194,195,196,197,198], including a wide range of nociception-related responses like reducing levels of inflammatory cytokines (IL-1β) and enhancing production of anti-inflammatory cytokines (IL-10), endogenous opioids, and BDNF [193]. Positive effects of EE on neuropathic pain were observed in a mouse model of chronic constriction injury (CCI) [194]. EE-mediated analgesic effects, reduction of depression-like phenotype, and memory deficits in the CCI mice were explained by involvement of neuronal PAS domain 4 protein and lowered levels of TNFα in the hippocampus [194]. EE also decreased stress-induced visceral pain and anxiety/depression-like phenotypes, while upregulating expression of IL-10 and downregulating expression of TNFα and IL-1β in specific parts of the mouse brain [199].

Pleiotropic effects of EE on pain-related physiology and behavior can modulate the activity of analgesic compounds. A combination of EE and ketamine was more effective than ketamine alone in reducing nociception in spinal cord injury model in rats [195]. Green LED light exposure enhanced the analgesic activities of morphine and ibuprofen in postsurgical pain model in rats [191]. Voluntary wheel running lowered doses of analgesic drugs needed to alleviate complete Freund’s adjuvant (CFA)-induced pain in mice [200]. The use of a running wheel to screen analgesic compounds was proposed [201]. The aforementioned studies suggest that EE containing running wheels, green light-emitting diodes, and music can improve the efficacy of drug candidates being evaluated for the treatment of pain.

## 6. Depression and Anxiety

Depression and anxiety are common mental health conditions that can impact an individual’s health-related QoL and can lead to disability and suicide [202,203]. Both disorders can affect mood, appetite, ability to engage socially, enjoyment of life, and the ability to take care of one’s self or their work. Depression and anxiety are treated with antidepressant and anxiolytic medications, as well as psychotherapies. Challenges with drug-based treatments are onset of action, non-adherence, drug-resistance, adverse effects, and abuse [204,205,206,207]. Challenges with psychotherapies, such as cognitive behavioral therapies (CBT), are accessibility, affordability, and effectiveness [208,209,210].

Digital health technologies are helpful for monitoring and treatment of anxiety and depression [211,212]. An early success in reducing depressive symptoms with a mobile app SuperBetter [213] and a computer game SPARX [214] opened doors to many mental health mobile apps [215,216]. Examples of DTx for depression include Deprexis^®^ [217,218,219], SparkRx^®^ [220], HelloBetter [221,222], and Daylight for anxiety [223]. Some mental well-being apps were shown to reduce depressive and anxiety symptoms in RCTs, for example Headspace [224], MoodHacker [225], or MoodGym [226,227]. In addition to mobile apps, VR-based apps are also effective in treating depressive and anxiety symptoms [228,229]. Most digital interventions for mental disorders employ such “active ingredients” as CBT, patient education, physical exercises, self-management, mindfulness practices, encouraging social interactions, and promoting healthy lifestyles [230,231,232,233,234]. Personalizing digital therapies for depression and anxiety is important to optimize their effectiveness [235,236]. Adjunct digital interventions for drug-based treatment of refractory depression appeared more effective, as compared to drug-alone treatment, illustrating the benefits of drug + digital combination therapies [237,238]. Opportunities to combine antidepressants with adjunct digital therapies were illustrated using software delivering non-pharmacological modalities shown to reduce depressive symptoms [239,240,241].

Preclinical testing of drug candidates and DTx “active ingredients” in EE-enhanced animal models for depression and anxiety can accelerate development of drug + digital combination therapies [242,243,244]. The need to innovate preclinical psychopharmacology through the “use of disease-relevant experimental perturbations” [245] was addressed by Branchi and colleagues who applied a drug-EE model for testing the efficacy of fluoxetine under either enrichment or stressful conditions [242,244]. Mice were exposed to interchanged stressful and EE living conditions, followed by 21-day treatments with either fluoxetine/EE or fluoxetine/stress [242]. Mice treated with fluoxetine/EE had significantly lower depressive symptoms, higher hippocampal and hypothalamic BDNF levels, and lower levels of cortisol compared to the “standard-cage” mice [242]. While fluoxetine and EE can reduce depression-like behaviors, they elicit distinct gene expression patterns in the amygdala, suggesting potential benefits of the fluoxetine/EE combination, instead of mono-therapies [246]. Another research group showed that EE reduced onset of action of a serotonin-norepinephrine reuptake inhibitor (SNRI) drug venlafaxine in mice, and these effects could be accounted for by parvalbumin interneurons in the hippocampus [247].

**Figure 8:**
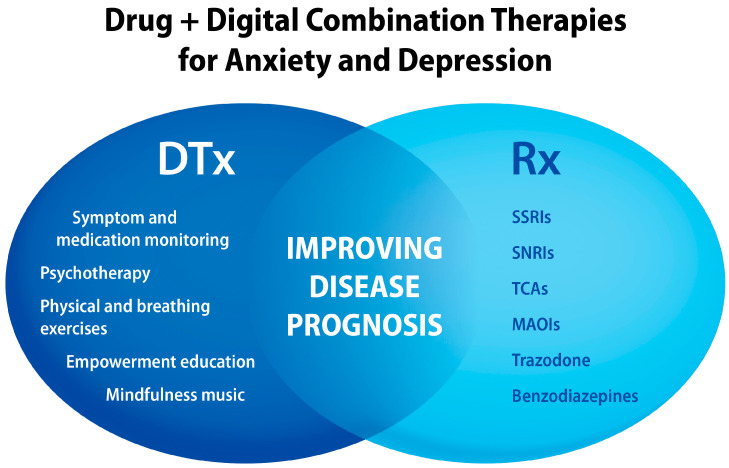
Examples of “active ingredients” delivered by digital health technologies that can be combined with antidepressants and anxiolytic drugs to improve therapy outcomes for mental disorders. Abbreviations: SSRIs, selective serotonin reuptake inhibitors; SNRIs, serotonin/norepinephrine reuptake inhibitors; TCAs, tricyclic antidepressants; MAOIs, monoamine oxidase inhibitors.

Animal studies show positive effects of EE-based treatments for depression and anxiety [248,249,250,251,252]. EE intervention in depression-induced male rat pups through administration of clomipramine reversed depression-like phenotype, depression-induced dentate gyrus hypotrophy, and basolateral amygdala hypertrophy [253]. Antidepressant and anti-anxiety effects of music were shown in chronic unpredictable mild stress in mice [252] and in a maternal separation rat model of early-life stress [254]. Anxiolytic effects of music were observed in knock-in transgenic mice (BDNF^Met/Met^) that exhibited fluoxetine-resistant anxiety symptoms [255]. Another DTx “active ingredient”, namely physical exercise, when tested in mice showed similar antidepressant and neuroregenerative effects as fluoxetine [256]. Physical exercise showed better outcomes than fluoxetine when comparing depressive behaviors and promoting hippocampal myelination [257]. From translational research and clinical perspectives, drug + digital combination therapies may offer improved effectiveness of psychopharmacology (Figure 8).

## 7. Epilepsy

Epilepsy is a neurological disorder characterized by patients having spontaneous epileptic seizures [258]. Epilepsy impacts cognitive and psychological functions, with higher prevalence of anxiety, depression, and migraine as comorbidities [259]. People with epilepsy experience higher incidence of body injuries, disability, diminished quality of life (QoL), and higher mortality rates [260]. Treatment options include antiseizure medications (ASMs) [261] and neuromodulation devices [262], while brain surgery remains an option for refractory epilepsy [263]. The multiple challenges with pharmacological management of epilepsy are drug resistance [264], drug adverse effects [265], medication non-adherence [266], polypharmacy [267], and drug shortages [268]. Notably, only 50% of newly diagnosed epilepsy patients become seizure free for one year, or longer, following their initial ASM treatment [269]. Given apparent limitations of ASMs, a rationale for integrating epilepsy self-management and pharmacological treatments via drug + digital combination therapies was proposed [13].

Mobile apps for people with epilepsy deliver self-management tools, including a seizure diary, medication reminders, stress and sleep management, patient education, and communication with a healthcare team [270,271,272]. An early example of online self-management programs was the WebEase platform which focused on medication, sleep, and stress management [273,274,275,276]. A 12-week RCT of a mobile app delivering medication reminders, seizure diary, healthy habits checklist (sleep, exercise, and stress), and health education showed increased medication adherence and self-efficacy [277]. EpApp is an epilepsy self-management app intended for adolescents, and it was shown to increase epilepsy knowledge and medication management; however, there was no significant difference in seizure burden after 4-week use [278]. In one RCT, the 6-month use of a self-management mobile app resulted in significant reduction of seizure frequency and improved self-management [279]. A web-based prototype DTx for the treatment of epilepsy was designed based on behavioral and music-based interventions that were previously shown to reduce seizures [22]. The “active ingredients” in this digital intervention included management of sleep, stress, and emotions; medication adherence; patient education; self-esteem; avoiding seizure triggers; and listening to specific music compositions [280,281,282,283,284,285].

Preclinical studies showed that EE and individual non-pharmacological interventions can reduce epileptic seizures in animal models of epilepsy [286,287,288,289]. EE intervention yielded disease-modifying (antiepileptogenic) effects by delaying an onset of seizures in a rat model of absence epilepsy [288]. These EE effects were transgenerational, since the next generation of the animals had reduced seizure frequency, as compared to the control offspring group [288]. Delayed kindling epileptogenesis via EE was observed in another rat model of epilepsy [290]. In post-status epilepticus TLE rat model of epilepsy, EE intervention was able to restore neurogenesis and cognitive functions and decrease the duration of spontaneous EEG seizures [291]. In the same TLE model of epilepsy, another group showed that EE reduced seizures and depressive symptoms [292]. In addition to preclinical findings on reducing epileptic seizures, EE was able to restore epilepsy-induced sleep and cognitive and behavioral impairments [293,294,295].

**Figure 9:**
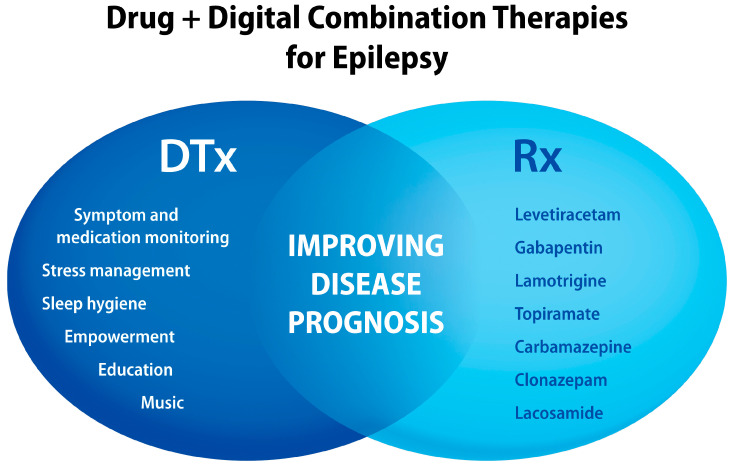
Examples of “active ingredients” delivered by digital health technologies that can be combined with antiseizure medications to improve therapy outcomes for people with epilepsy, including those with refractory epilepsy.

A promising DTx “active ingredient” for epilepsy is specific music [280,281,296,297,298,299,300,301], and the clinical effects were also reproduced in preclinical studies [23,302,303]. Xu and colleagues showed that exposure of TLE mice to a specific music composition enhanced the anti-seizure activity of sub-effective doses of valproic acid or levetiracetam [303]. In the corneal kindling mouse model of epilepsy, the same music composition reduced cumulative seizure burden and mortality rates in the music-treated group [23]. In the spontaneous absence epilepsy rat model, music exposure reduced both seizure frequency and spontaneous high-rhythmic spike discharges [302]. Another possible DTx “active ingredient” for epilepsy management is physical activity [304,305,306,307]. Preclinical studies show that physical exercise can reduce epileptic seizures [308,309,310] and enhance the efficacy of ASMs, such as carbamazepine and valproate [311,312]. Translational aspects of physical exercises in epilepsy suggest such benefits as antiepileptogenesis and neuroprotection [313,314]. As illustrated in Figure 9, the combination of ASMs with digitally delivered non-pharmacological modalities can offer better seizure control, as compared to drug-alone interventions.

## 8. Other Indications and Applications

Drug + digital combination therapies can benefit people living with cardiometabolic disorders. Digital therapies for the treatment of diabetes type 2 were pioneered with the development of DTx BlueStar^®^ [5,315,316], and showed efficacy in reducing HbA1c [317,318]. Diverse diabetes digital health technologies include such DTx as glucose tracking/monitoring systems apps, self-management, and lifestyle support apps (e.g., d-Nav, Glooko, mySugr, Dexcom, and Dario) [319,320]. New opportunities exist to combine DTx with automated close-loop insulin delivery systems [321]. DTx for hypertension and obesity can be integrated with beta blockers to improve blood pressure management, or with semaglutide (Ozempic^®^, Wegovy^®^) for weight loss, respectively [322,323,324].

Drug-based management of chronic infections (e.g., dolutegravir for HIV/AIDS, or sofosbuvir for hepatitis C) can be integrated with DTx that improve therapy outcomes through medication tracking and diverse self-management interventions [325,326,327]. Notably, positive effects of EE and physical exercise on the innate and adaptive immune functions and viral infections in rodents were reported [328,329,330], opening translational opportunities to develop combination therapies for chronic infections [331].

Gene therapy is another example where combinations with DTx can improve therapy outcomes. Since gene therapies aim to improve symptoms after only one injection, developing DTx as an adjunct digital intervention or/and “biologic + digital” combination product may support a patient’s journey before and after the correction of a mutated gene. In the case of the treatments for amyotrophic lateral sclerosis with tofersen [332] and spinal muscular atrophy with onasemnogege abeparvovec [333], these patients could use digital technologies for monitoring therapy outcomes and delivering neurorehabilitation exercises [334,335,336,337]. “One-time treatment” gene therapies for indications where self-management and self-efficacy can improve therapy outcomes can be developed together with DTx that provide clinically meaningful benefits beyond the injection of DNA vectors [338].

**Figure 10:**
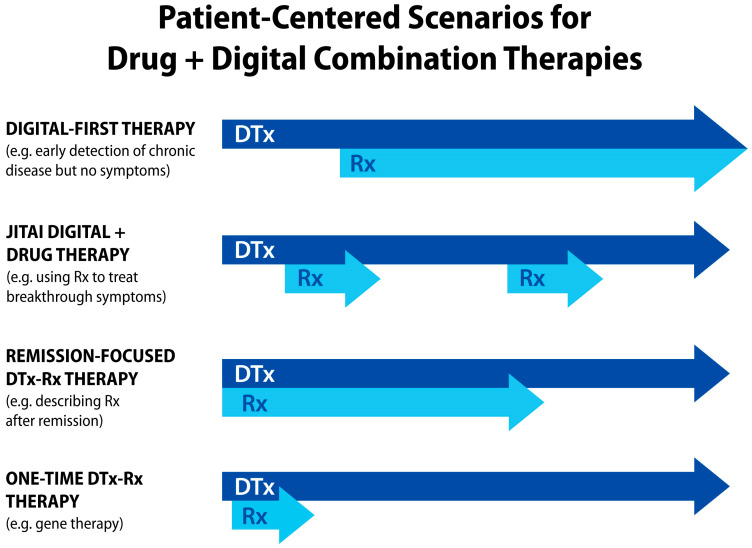
Examples of patient-centered scenarios for drug + digital combination therapies that offer personalized and just-in-time adaptive interventions (JITAIs) depending on a patient’s disease prognosis, including disease symptoms and biomarkers (both physiological and digital).

Due to software flexibility in delivering just-in-time adaptive interventions through DTx, drug + digital combination therapies can redefine precision medicine by providing digital therapy content tailored to a patient’s needs and disease progression [14]. Given advances in biomarker research for metabolic or neurodegenerative conditions, drug + digital combination therapy can start with digital-first care [339,340]. This approach is applicable for the treatment of osteoarthritis [341,342]. For rheumatoid arthritis and other chronic inflammatory conditions associated with flares [343], drug + digital combination therapies offer the flexibility of tapering DMARDs between longer periods of remission. For people living with chronic pain or depression, personalized drug + digital combination therapies can adjust drug-based management of symptoms after remission. Figure 10 illustrates diverse scenarios for patient-centered care in which pharmacotherapies are adjusted based on a disease activity status and prognosis.

**Figure 11:**
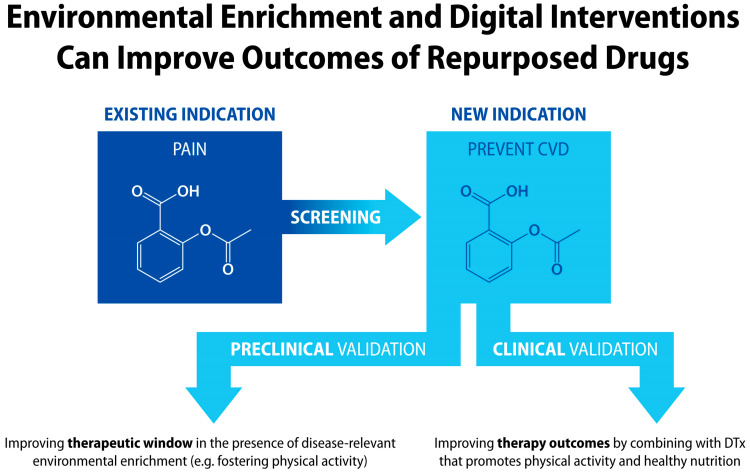
Aspirin as an example of drug repurposing from pain to prevention of cardiovascular diseases. During discovery and validation of drug-repurposing candidates, preclinical studies in the presence of EE relevant to a new disease indication can improve therapeutic window (TD_50_/ED_50_, where TD_50_ is the median toxic dose, and ED_50_ is the median effective dose). Integration of a repurposed drug with disease-specific digital interventions via drug + digital combination therapy can further improve patient outcomes.

Another application of drug + digital combination therapies and products is drug repurposing, which is recognized as an innovative way to expand indications for existing drugs [344,345,346]. Computational, molecular, and cellular screening approaches aim to match drug phenotypes with a new therapeutic target. Once a new indication is identified, preclinical validation of a repurposed drug in a new target disease animal model can include both the “standard” testing conditions, as well as the EE conditions that include disease-relevant surrogate ingredients for DTx (Figure 11). Similarly, adjunct digital intervention during clinical validation of a repurposed drug may offer better primary endpoint outcomes, since such combination therapy can include new disease-specific self-management digital content. Example applications for chronic conditions include repurposing anti-inflammatory drugs for cardiovascular [347], psychiatric [348], neurological [349], and autoimmune disorders [350].

## 9. “Patent Cliff” as an Incentive for Developing Drug + Digital Combination Therapies

Opportunities to develop drug + digital combination therapies can be illustrated by adalimumab (Humira^®^) indicated for rheumatoid arthritis and other autoimmune and inflammatory disorders. While facing a “patent cliff” for this commercially successful biologic and competition from several FDA-approved biosimilars, AbbVie engaged with diverse strategies to extend the US market exclusivity beyond 2023 [351,352]. However, Humira-based treatments have not been innovated by developing drug–device combination products comprising adalimumab and DTx that could provide additional clinical benefits [79,80,81]. Such a drug + digital combination product approach could offer copyright-protected therapy that could improve both Humira’s market dominance and patient outcomes. Instead, AbbVie continues to offer a mobile app “Humira Complete”, delivering medication reminder, injection instructions, symptom trackers, creating personal goals, and connecting with a Nurse Ambassador, among other features.

Transforming the “Humira Complete” app into DTx would require (1) expansion of disease self-management and empowerment interventions and (2) clinical validation of its efficacy in RCT. The FDA’s draft guidelines illustrate innovative opportunities for marketing adalimumab *plus* a mobile app for which “use of the prescription drug use-related software with the product results in a meaningful improvement in a clinical outcome as compared to use of the product without the prescription drug use-related software” [11]. Therefore, after ending the market exclusivity, brand-name Humira^®^ may compete with cheaper and more effective biosimilars seamlessly integrated with DTx that would deliver clinically meaningful benefits.

**Figure 12:**
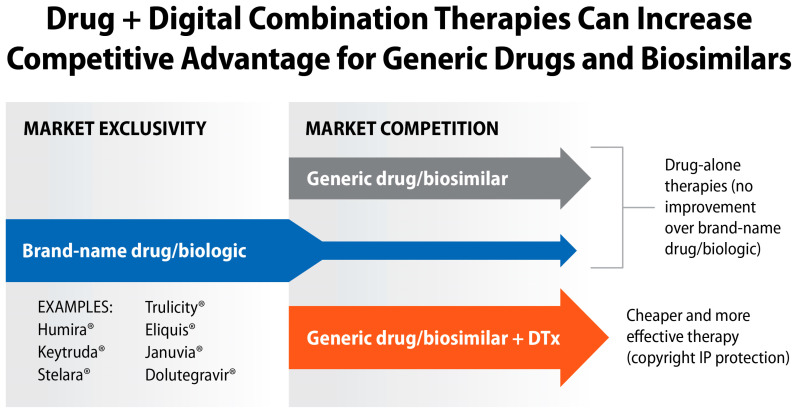
The role of DTx in innovating pharmacotherapies after brand-name drugs/biologics lose the market exclusivity due to patent protection. Selected examples of blockbuster drugs that face “patent cliff” by 2030.

As shown in Figure 12, these opportunities to advance drug + digital combination products apply to many blockbuster drugs that face a “patent cliff”. Pharma and biotech companies that own patent-protected market exclusivity for brand-name drugs and biologics can face new challenges when *more effective* combination therapies with respective generics enter patient-driven competition. We hypothesize that anticipation of marketing “more innovative” drug–device combination products from generic drug competitors will motivate development of drug + digital combination therapies.

## 10. Limitations of This Review

While this review is focused on translational aspects of drug + digital combination therapies, it has several limitations, including (1) a lack of reviewing research on mechanisms of action (MOA) of digital and EE interventions, (2) restricting overview of existing studies to only several chronic diseases, (3) not discussing regulatory aspects, patient privacy and security protections, interoperability, standards, and cost-effectiveness considerations of DTx, and (4) literature selection bias of a narrative review. Pleiotropic MOA of EE interventions was reviewed elsewhere [193,353,354]. It is also noteworthy that a diversity of EE experimental protocols precludes generalization for MOA [355]. A lack of data for physiological outcomes of DTx interventions is likely due to an initial focus on the efficacy studies rather than to delineate MOA. Regarding the second limitation of this review, we acknowledge that drug + digital combination therapies are applicable to other chronic conditions not discussed here. For example, advances in DTx for Parkinson’s disease, including the MedRhythm’s technology [356], support their combinations with levodopa. Digital interventions for bipolar disorder are developed by MindPax and others [357,358]. DTx for insomnia, such as Somryst^®^, Sleepio^®^, Somzz^®^, and HelloBetter^®^ Sleep, can be integrated with drug-based treatments for sleep [359].

Regulatory aspects for DTx and drug + digital combination products have been omitted in this review, due to the complexity of evolving regulations across the FDA, EMA, and other country-specific agencies [6]. The FDA draft guidelines support integration of mobile apps with prescription drugs and biologics, opening a new frontier for pharma and biotech to advance drug + digital combination therapies. Cost-effectiveness studies support financial benefits of DTx [360,361,362]. However, there are also multiple barriers to a broader implementation of DTx in healthcare systems [88,363]. Insights from early adopters of DTx, for example Germany, can be helpful for healthcare stakeholders in other countries to navigate both opportunities and challenges of bringing digital and drug + digital combination therapies to patients [364].

This narrative review also has the innate limitation of summarizing relevant articles without the rigor of a systematic review. An apparent selection bias can impact both an objective analysis of published literature for individual chronic diseases, and the validity of the authors’ conclusions.

## 11. Conclusions

Clinical and preclinical studies support translational research on integrating digital interventions with pharmacotherapies. Available evidence for digital interventions varies from disease to disease while showing clinically meaningful benefits for patients living with the chronic diseases reviewed here. Academic and industry groups focused on drug discovery and preclinical development may consider evaluating their lead compounds in the presence of DTx “active ingredients” delivered as EE intervention, hence increasing the odds of advancing IND candidates to clinical studies. When studying new compounds in animal disease models, this “EE-pharmacology” approach will require more standardized testing conditions. The observed diversity in experimental design in animal studies of EE + drug interventions warrants establishing preclinical guidelines for investigating DTx “active ingredients” that support future co-development of drug + digital combination therapies.

Developing drug + digital combination therapies is still in its infancy, despite apparent opportunities to improve effectiveness of pharmaceutical drugs and biologics using digital interventions [12,13,22,23]. In conclusion, a quote from Helen Keller, “Alone we can do so little; together we can do so much”, can serve as encouragement for translational and clinical research to develop drug + digital combination therapies, including drug–device combination products for a personalized treatment of chronic diseases.

## Data Availability

Not applicable.
